# The Rise and Fall of Financial Flows in EU 15: New Evidence Using Dynamic Panels with Common Correlated Effects

**DOI:** 10.1007/s10614-023-10366-7

**Published:** 2023-03-16

**Authors:** Mariam Camarero, Alejandro Muñoz, Cecilio Tamarit

**Affiliations:** 1 Department of Economics, University Jaume I and INTECO, Campus de Riu Sec, 12080 Castellón, Spain; 2 Department of Applied Economics II, University of València, Av. dels Tarongers, s/n Eastern Department Building, 46022 Valencia, Spain; 3 Department of Applied Economics II, University of València and INTECO, Av. dels Tarongers, s/n Eastern Department Building, 46022 Valencia, Spain

**Keywords:** Capital mobility, Feldstein–Horioka puzzle, Structural breaks, Cross-sectional dependence, Cointegration, unit roots, F36, F45, O16

## Abstract

This paper assesses capital mobility for a panel of 15 European countries for the period 1970–2019 using dynamic common correlated effects modeling as proposed in Chudik and Pesaran (J Econ 188(2):393–420, 2015). In particular, we account for the existence of cross section dependence, slope heterogeneity, nonstationarity and endogeneity in a multifactor error correction model (ECM) that includes one homogeneous break. The analysis also identifies the heterogeneous structural breaks affecting the relationship for each of the individual countries. The ECM setting allows for a complete assessment of the domestic saving-investment relationship in the long-run as well as two other elements usually neglected: short-run capital mobility and the speed of adjustment. When we account for a single homogeneous break, this is found at the euro inception. We obtain that long-run capital mobility is high but not perfect yet. We also provide empirical evidence for the Ford and Horioka (Appl Econ Lett, 24(2), 95–97, 2017)’s hypothesis, who argue that goods market integration is a necessary condition to obtain zero correlation between domestic saving-investment. Our results stress the role played by the euro as a booster for both financial and real integration. However, a complete degree of economic integration has not been fully achieved. Short-run capital was highly mobile for the whole period, with some exceptions, coinciding with turmoil episodes. Additionally, from the application of the CS-DL threshold analysis proposed by Chudik et al. (Adv Econ, 36, 85–135, 2016), we find that economic risk and openness play a key role in capital mobility.

## Introduction

Capital mobility is a key issue in international macroeconomics. Baele et al. (2004) outline three widely accepted interrelated benefits of financial integration: higher efficiency, better opportunities for risk sharing, and more potential for growth. Financial liberalization allows capital to be reallocated where it is more efficient and eventually, this process improves welfare and growth. Moreover, integrated banking and capital mobility do provide broader protection from shocks in a single currency area and enhance “cross-country risk-sharing”, that is, it helps to decouple consumption and output dynamics when an asymmetric shock occurs (Kalemli-Ozcan et al., 2003).^1^ In order to take advantage of these positive effects, barriers to capital mobility started to be removed by the 1970s in the US and the UK, followed by other developed countries during the 1980s.

In the EU, this liberalization process also started in the 1980s and the beginning of the 1990s following the Single Market initiative, that implied full capital mobility by 1992 after the Maastricht Treaty (OECD, 2011). Later, the process followed with the creation of the European passport for financial services and the Financial Services Action Plan starting in 1999, the Lamfalussy process from 2001, and the Larosière Report in 2009 (Larosiere, 2009), which enshrined the vision of a single rulebook and resulted in the creation of the European supervisory authorities.

However, since the Great Recession of 2007–2008, financial markets have experienced a striking dichotomy regarding the on-going process of globalization. In the specific case of Europe, there has been a remarkable process of fragmentation since the beginning of the crisis and the EU is now fostering financial integration through the creation of a banking and capital markets union (CMU hereafter). The Commission adopted the first CMU action plan in 2015. Since then, the EU has made significant progress to put the building blocks in place towards increasing financial integration.^2^ Therefore, the analysis of the evolution of the degree of financial integration in the EU has regained momentum.

Despite the welfare improvement caused by capital mobility across countries, since the 1990s, there has been a side non-desired effect in terms of increasing and persistent external current account imbalance, as well as in its financial counterpart. That process is not constrained to the Euro area (EA); on the contrary, it has occurred in parallel with a process of globalization characterized by the removal of capital barriers around the globe. Different hypotheses have come forward, trying to explain such external imbalances; one of the most popular is the “savings glut” hypothesis, formulated by Bernanke (2005) and extended in Horioka et al. (2016). According to this hypothesis, external disequilibria are caused by macroeconomic imbalances between national savings and investment (*S* and *I*, respectively, hereafter) in a context of financial liberalization rather than by industrial, commercial, or exchange rate policies. Persistent imbalances between national *S* and *I* would be at odds with the existence of a Feldstein–Horioka puzzle and would lead to the creation of increasing external disequilibria in the following way: large surplus countries, where $S>I$, contribute to the world-saving pool; such excess of saving causes the real interest rate to decrease, which allow the access of deficit countries to new credit, pumping larger external disequilibria. However, this process sharply ended with the 2007 financial crisis and the subsequent adjustment that took place afterward.

The above re-balancing procedure can be especially costly within a monetary union, as the EA. The financial sudden stop inside the EA created an asymmetric shock, as countries were affected differently depending on their relative degree of external imbalance (Baldwin and Giavazzi, 2016). At the core of the problem lies an issue of balance of payments adjustment within a monetary union.^3^ Unwinding these imbalances led to sharp increases in sovereign debt and induced a sovereign-bank feedback loop. It also created spillover effects across the Member States that endangered the stability of the EA as a whole and marked a period of economic and financial divergence among the Member States. As a consequence of the limited cross-border financial integration in banking and capital markets, significant differences in financing conditions between EU countries arose during the crisis, slowing the recovery and undermining economic convergence. The current Covid-19 crisis will only complicate more this scenario with increasingly uncertain external relations across countries.

In this paper, we assess capital mobility for the EU-15 between 1970–2019 in the context of the definitions of Feldstein and Horioka (1980) and Ford and Horioka (2017). Moreover, we focus on the impact that the different stages of financial and real integration have had on capital mobility. Obviously, a reliable and econometrically robust measure of the degree of capital mobility along time, such as an estimate of the savings-retention coefficient, is needed for this purpose. We argue that the high saving-investment correlations found in seminal works were due to the neglect of the non-stationary properties of the series and failure to account for structural breaks in the long run relationship between the variables. Moreover, the analysis of an economic integrated area as the EA can be improved using panel data, as it enriches the information included in the analysis. However, panel data present a series of econometric issues, many times neglected in empirical applications, such as the existence of observed and unobserved common effects, cross-section dependence, parameter heterogeneity, and endogeneity in a multifactor dynamic error framework.^4^ Precisely, the salient feature of our econometric approach is that it allows us to exploit both the cross-section and time information included in T-large panels accounting for all these issues commonly disregarded in previous literature. We follow an empirical approach that allows for a complete analysis of capital mobility, looking at four dimensions: first, the long-run relationships; second, the path followed to return to long-run equilibrium when a shock occurs; third, the presence of structural breaks affecting the relationship and lastly, the short-run impact of domestic saving on domestic investment, an effect usually disregarded in this literature. To the best of our knowledge, there are no studies that jointly address all these elements in the analysis of capital mobility and the FH puzzle. To this aim, we propose an econometric strategy in two stages. First, we examine the long-run and short-run links between domestic investment and saving using Chudik and Pesaran (2015) dynamic common correlated effects (DCCE hereafter) modelled as an error correction model that allows for one structural break. In a second stage, using the Cross-sectional Distributed lags (CS-DL hereafter) proposed in Chudik et al. (2016) we assess how the long-run relationship between domestic investment and saving changes when risk and economic openness reach a certain threshold. The DCCE is a modified estimator well suited for handling dynamic and heterogenous coefficients of a panel model that incorporates lagged dependent and weakly exogenous regressors.

This paper is organized as follows: in Sect. 2, we review the empirical literature on the subject and present the theoretical background on capital mobility using the FH approach. Section 3 summarizes the data and the methodology used in this paper. Section 4 discusses the empirical results. Finally, section 5 concludes.

## Related Literature

### Capital Mobility: Price Versus Quantity Approach

The measurement of the degree of capital mobility is essential to assess the state of the global economic environment. In good times, international financial flows channel savings to the countries and regions of the world where they are most productive, while during crises, they have the potential to disrupt the financial systems of the most vulnerable countries and therefore constitute a key factor for global financial instability. However, measuring capital mobility is a challenging task. Financial market integration implies that assets with identical risks and returns should be priced identically regardless of where they are transacted. However, to get this result, a series of different conditions should be met, and the literature has traditionally considered two approaches.^5^ While the first one is known as the *price approach* and focuses on the co-movements between domestic and foreign rates linked by the exchange rate, the second approach - also known as the *quantity approach* - studies the co-movement of the variables that directly materialize capital mobility, that is, investment and savings.^6^.

Concerning the price-approach,^7^ there are three accumulative criteria that have been posited in the literature. The first criterion (capital mobility of type I) is the “covered nominal interest rate parity” or CIP. If the CIP is fulfilled, there would be no country premium. If *i* and $i^*$ stand for nominal domestic and foreign interest rates and *F* and *E* are, respectively, the forward and spot exchange rates, the expression $i_{t}-i^{*}_{t}-(F^{t+j}_t-E_{t})=0$, means that the nominal interest rate differential should be compensated by an equal gap between forward and spot nominal exchange rate.

The second criterion is the “uncovered nominal interest parity” or UIP. It implies the fulfillment of type I mobility plus zero exchange risk premium. If this criterion holds, both country premium and exchange risk premium are zero, $(F^{t+j}_t-E_{t})-(E^{*e}_{t+j}-E_t)=0$, and therefore, perfect capital mobility of type II holds.

The third criterion is the “real interest parity” or RIP. This is the most restrictive definition of capital mobility as it requires criteria I and II to hold plus zero expected real depreciation. If this criterion, also called capital mobility of type III, is satisfied, it implies perfect financial and non-financial capital mobility, i.e. zero country premium, zero exchange risk premium plus zero expected real exchange rate change $$E_{t+k}^e-E_t=\pi _t^e-\pi _t^{*e}$$. Therefore, domestic and foreign real interest rates are equalized $$ r_{t+j}^e-r_{t+j}^{*e}=0$$.

Finally, according to this taxonomy, the strongest and more complete definition of capital mobility is given by the quantity-based approach, that is, the Feldstein–Horioka condition: it requires criteria I, II and III to hold, implying zero correlation between domestic investment and saving. This means that an exogenous change in the national saving rate should have no effect on the domestic investment rate.

### FH Condition for Financial Integration

Feldstein and Horioka (1980) estimated using OLS the relationship between the ratios of saving and investment over GDP for the period 1960–1974, as well as for three subperiods (1960–1964, 1965–1969 and 1970–1974). In particular, they estimate the following equation:

1 $$\begin{aligned} \left( \frac{I_{i}}{Y_{i}}\right) =\alpha +\beta \left( \frac{S_{i}}{Y_{i}}\right) \end{aligned}$$

where $({I_i}/{Y_i})$ is the ratio of gross domestic investment to GDP and $({S_i}/{Y_i})$ is the ratio of gross domestic saving to GDP. Coefficient $\beta$ is called the saving-retention coefficient, which measures how changes in the national investment ratio are explained by exogenous changes in the domestic saving ratio.

The authors argue that in a world with perfect capital mobility, $\beta$ should be zero for small countries, whereas it would represent their share of the world capital stock for large countries. The reason for $\beta$ to be zero is that in a world with fully integrated capital markets, any exogenous variation in domestic savings should not affect domestic investment, arguing that capital would move to the countries with the highest return. In contrast, in a closed economy, the saving-retention coefficient should be 1 because the domestic savings accommodate all variations in domestic investment.

Feldstein and Horioka (1980) found a $\beta$ coefficient of 0.88. They argued that this coefficient implied that international capital mobility and the degree of integration of the international capital markets were low. The FH result has been tested in many papers. However, their result has remained robust in the cross-section analysis. This result confronts what international macroeconomics predicts in a process of globalization. This is why the FH result has given rise to the “Feldstein–Horioka puzzle”(Feldstein and Horioka, 1980).

### Assumptions Behind the FH Regression

From a theoretical point of view, for the FH regression to be valid some assumptions should be satisfied. The FH condition for capital mobility is the strongest one, and it requires the other three criteria plus three strong assumptions:

*First:*

2 $$\begin{aligned} \left( \frac{I_{i}}{Y_{i}}\right) =\beta _0-\beta _1r_i+u_i \end{aligned}$$

where (2) implies that the investment rate depends on the real interest rate and an unknown error term (that is, other factors that affect the investment rate differently from the domestic real interest rate).

*Second:*

3 $$\begin{aligned} Cov\left( u_i,\frac{S_i}{Y_i}\right) =0 \end{aligned}$$

(3) implies that for the saving-retention coefficient to be unbiased, none of the determinants of the investment rate must be correlated with the saving ratio in country i. If this strong assumption does not hold, it would give rise to endogeneity problems. In fact, it is sensible to think that both rates are affected by population or productivity shocks.

Third:

4 $$\begin{aligned} r_i=r^* \end{aligned}$$

Assuming that the real interest parity holds, then (4) implies that the country is unable to affect the world real interest rate, that is, $r^{*}$ is exogenous.

Therefore to obtain a zero saving-retention coefficient in equation (2) the world interest rate $r^*$ has to be exogenous as well as the error term in equation (3) should not be correlated with the saving rate.

Ford and Horioka (2017) argued that the real interest parity does not need to hold (the third assumption above). Instead, they claimed that the integration of both goods and financial markets is necessary to obtain a zero coefficient in the saving retention parameter.

### Assessment of Capital Mobility: Still a Debate

After the findings of Feldstein and Horioka (1980), many papers have looked for factors impeding capital mobility. Niehans (1986) argues that the removal of barriers to capital transfers does not ensure its mobility across countries. In the same vein, Obstfeld and Rogoff (2000) developed a model with transaction costs for international trade in goods finding that the sole existence of frictions in the good markets might prevent capital mobility across countries. More recently, Ford and Horioka (2017) maintain that financial markets integration is not a sufficient condition to achieve capital mobility, i.e. they state that FH’s results can be due to the absence of globally integrated goods markets, and hence, both financial and goods markets integration are needed for capital mobility to existing. Eaton et al. (2016), have underlined that the net transfers of capital among countries depend not only on the integration of the financial markets but also on the integration of goods and services market. This evidence is also in line with the joint fulfillment of the UIP and the purchasing power parity (PPP) conditions as shown in Johansen and Juselius (1992), Juselius (1995), Camarero and Tamarit (1996), and more recently, in Pesaran et al. (2000).

In this sense, our EU sample is a natural test for capital mobility because there are no barriers either to capital or to goods mobility and there has been a gradual integration process that substantially decreased financial and trade frictions. In case capital mobility remains persistently low, we should conclude that there are other reasons for this result, probably the so-called “home bias”. Following Ford and Horioka (2017), we study whether the process of economic integration (financial and real) in Europe has encouraged capital mobility.^8^

### Attempts to Solve the FH Puzzle

The FH puzzle (Feldstein and Horioka, 1980; Feldstein, 1982) has given rise to extensive literature trying to solve it. This literature can be classified into three categories.^9^

The first one seeks to reconcile the large correlation found between domestic saving and investment with the existence of high capital mobility constructing new theoretical models. One strand of the literature highlights the role of the intertemporal budget constraint for the explanation of the FH puzzle (Sinn, 1992; Jansen, 1997, 1998, 2000; Obstfeld and Rogoff, 1995; Coakley et al., 1996; Shibata and Shintani, 1998). An extension of that argument is related to the government interventions to offset private net capital flows (Fieleke, 1982; Tobin, 1983; Westphal, 1983; Summers, 1988).

The second line of research includes those papers that do not agree with Feldstein and Horioka (1980) results. One of the most important criticisms has to do with the choice of the countries included in their paper as this could be a source of a *sample selection problem*: as FH only include large developed countries (LDC), this may cause an upward bias on the saving-retention coefficient. Savings shocks that hit large economies are expected to affect the world interest rate and hence their domestic investment. Therefore, we may expect a higher saving-investment correlation for large economies. Harberger (1980), using a group of small and large countries, finds that the former have experienced stronger capital inflows and outflows than large economies. Those results were also supported by Sachs (1983) and Murphy (1984). Later, Tesar (1991) excluded Luxembourg from her sample and found that the saving-retention coefficient changes from 0.35 to 0.84, concluding that there is a problem of sample selection bias. A second, criticism of the results is due to Tobin (1983). He argues that the FH regression suffers from *endogeneity problems* that should be properly addressed.^10^ Later studies have supported his view, such as Dooley et al. (1987), Bayoumi (1990), Tesar (1991) and Kasuga (2004). The third source of criticism is related to the *non-stationary properties of the variables*. Jansen and Schulze (1993) and Coiteux and Olivier (2000) argue that if the variables are I(1) and they are in levels the result may not be valid. To solve this problem, Bayoumi (1990) introduces the variables in first differences. Finally, Obstfeld and Rogoff (1995) and Ho (2000) argue that time-averaged data in cross-section studies tend to overestimate or underestimate the value of the saving-retention coefficient.

Relying on the previous objections, the empirical literature has focused on improving the econometric techniques in order to overcome previous pitfalls. We can classify the papers according to the statistical methods depending on the data used: cross-section, time series, and panel data methodologies. First, concerning *cross section data*, Murphy (1984) estimated the saving-investment relationship for a group of 17 countries finding that the correlation was higher for large countries and relating the FH results to country size instead of capital immobility. Feldstein (1982) updated FH paper by extending the sample from 1960–1979 with no change in the results. Later, Feldstein and Bacchetta (1991) using a sample of 23 OECD countries found that the saving-retention coefficient had marginally declined over time. Other studies have used different samples and time periods, but within the cross-sectional setting, the FH result has remained robust.

As for *time series studies*, Feldstein and Horioka (1980) also ran the FH regression using annual data for 21 countries finding an average saving-retention coefficient of 0.64. Obstfeld (1986) obtained a saving-retention coefficient from 0.13 to 0.91. Tesar (1991) and Frankel (1991) also used time series and the latter showed that the saving-investment correlation had fallen from the 1980s in the US. Also in this literature, Miller (1988) studies the existence of cointegration between saving and investment over the period 1946–1987 finding that there only was cointegration under a fixed exchange rate regime. Kejriwal (2008) revisits the FH puzzle for 21 OECD countries. He finds evidence from 1 to 3 regime changes. Ketenci (2012) and Camarero et al. (2021) apply the same econometric methodology than Kejriwal (2008) to the EU. The latter study the period 1970–2019 and find a downward trend in the saving-investment retention since the 70 s for the so-called “core countries” and identify several breaks coinciding with relevant economic integration episodes.

However, to analyze economic areas, the use of panel data can be more suitable. Indeed, panel data provides more information, variability, efficiency and it minimizes estimation biases compared to pure cross-sectional and time series data. Examples of applied papers testing the FH relationship are: Coakley et al. (2004), Krol (1996), Coakley and Kulasi (1997), Corbin (2001), Dash (2019), Narayan (2005) and Kim (2001) with similar results than those found using time-series. However, these studies do not take into account structural breaks in the long-run saving-investment relationship. This issue is tackled by a few number of papers: Westerlund (2006) finds more evidence of cointegration between saving and investment once he accounts for structural breaks in the panel. Katsimi and Zoega (2016) found that the breaks coincide with the European single market in 1993, the euro in 1999 and the 2008 financial crisis. Kumar (2015) associates structural breaks with regional agreements and finds that these agreements have encouraged capital mobility.^11^ An alternative solution to abrupt structural breaks is to allow for the parameter in the FH regression to vary over time. This is the approach, among others, adopted by Telatar et al. (2007), Gomes et al. (2008), Ma and Li (2016), or Khan (2017). More recently, Camarero et al. (2020) develop a time-varying parameters state-space model that they apply to a panel of EU countries.

Another potential problem of the traditional panel methods is that they are built under strong cross-sectional independence assumptions. Several studies have addressed this issue. First, Byrne et al. (2009) decompose idiosyncratic and global components of the long-run saving-investment relationship and find that the global components in saving and investment react to global shocks. Giannone and Lenza (2010) propose the use of factor-augmented panel regressions to isolate idiosyncratic sources of fluctuations so that countries are allowed to react with specific sign and magnitude to global shocks. Such heterogeneous propagation mechanism of global shocks yields saving-retention coefficients that drop from the 1980s. In the same vein, Costantini and Gutierrez (2013) found that, once global shocks are taken into account through common factors, the FH puzzle disappears. Finally, Murthy and Ketenci (2020) use Pesaran (2006)’s CCEMG and Chudik and Pesaran (2015) DCCEMG tests to investigate the FH puzzle for a panel of 27 African countries.

Focusing on the European case, Drakos et al. (2017) and Drakos et al. (2018) assessed the correlation between domestic saving and investment for 14 EU countries employing panel data techniques for the period 1970–2013 and 1970–2015, sequentially. In the former paper, they obtained a sizeable saving-retention coefficient suggesting that this result is consistent with the intertemporal budget constraint. They concluded that the speed of adjustment towards the long-run equilibrium showed some sign of capital mobility. However, the speed of adjustment obtained for Greece (2 years), Italy (2 years), Portugal (4 years), and Spain (4 years) did not support the large and persistent external imbalances observed for these countries.^12^ In the latter paper, they took into account cross-sectional dependence and found a moderate degree of capital mobility and that the FH puzzle was partially present. However, both papers failed to consider structural breaks, which may lead to an underestimation of the degree of capital mobility. But and Morley (2017), applying panel techniques for a sample of OECD countries (some of the EA members), found that the correlation between domestic saving and investment dropped to record levels right before the 2008 financial crisis, and the puzzle returns afterward. Their results also suggest that the puzzle for net capital-importing and capital-exporting countries differs. By contrast, Ketenci (2018) found an insignificant impact of the 2008 financial crisis on the level of capital mobility. Camarero et al. (2020), using time-varying parameters techniques with panel data, found that the FH coefficient declines over time, confirming an increase in capital mobility and international openness.

Finally, the ECB performs periodical studies on the degree of financial integration in the EA.^13^ The ECB uses two approaches. First, in ECB (2018), following the two seminal papers of Lewis (1995) and Asdrubali et al. (1996), the ECB examines the evolution of cross-country risk-sharing in the EA and finds that risk-sharing is still low and unstable, with an increase in the correlation between consumption and output dynamics after the financial and sovereign crisis. Second, ECB (2018) analyzes the degree of financial integration in the EA and the fulfillment of the FH puzzle. They augment the usual FH equation with country-specific variables to account for global shocks affecting those economies. However, the report highlights that the estimation is highly volatile making the interpretation of the results difficult, which calls for further research in the area.

## Methodology

### Description of the Methodology and Data

In this section, we describe the econometric methodology applied in this paper. We divide our analysis into two parts. In the first one, we examine the long-run and short-run links between domestic investment and saving using Chudik and Pesaran (2015)’s dynamic common correlated effects (DCCE) estimator. Moreover, we obtain an error correction model allowing for one common structural break. In the second part, we examine how the long-run relation between domestic investment and saving changes when risk and economic openness reach a certain threshold. For this, we apply cross-sectional distributed lags (CS-DL), as proposed by Chudik et al. (2016). Sequentially, the econometric procedure is as follows. First, we apply Pesaran (2020) test to assess the existence of cross-sectional dependence not only on the variables but also on the saving-investment regression. Second, once the existence of dependence has been tested, we determine the order of integration of the variables using Bai and Carrion-i Silvestre (2009) unit root test with cross-sectional dependence and structural breaks. In a third step, we test for cointegration between domestic savings and investment by applying Banerjee and Carrion-i Silvestre (2015) approach. As in the case of the unit root test, the Banerjee and Carrion-i Silvestre (2015) cointegration test allows for structural breaks and cross-sectional dependence. Using this approach, we identify and analyze the heterogeneous breaks that affect the domestic I-S relationship for all the panel members. For comparability, we also apply the cointegration test by Banerjee and Carrion-i Silvestre (2017) that takes into account cross-sectional dependence using common correlated effects (CCE hereafter) introduced by Pesaran (2006). Finally, we estimate a panel error correction model augmented by the cross-sectional averages and their lags, based on the DCCE with a common structural break (Chudik and Pesaran, 2015). Then, we study the effect of economic risk and openness on the FH relationship when a certain threshold is reached using CS-DL (Chudik et al., 2016).

As our interest is focused on the long-run and short-run impact of the savings-investment relationship in the EU, the role of economic integration, and how economic risk can affect capital mobility in the EU, we have tried to obtain the largest panel available with annual data. In our case, we have worked with the EU15^14^ and the source of the data is the OECD database for the period 1970–2019.

Regarding our data, we measure investment with gross fixed capital formation, whereas, for saving, we use “basic saving”, as recommended by Baxter and Crucini (1993). This variable is defined as GDP minus total consumption (both public and private); both variables are expressed as a percentage of GDP. To measure the overall economic openness for the EU 15, we use the average KOFEcGI index (financial and trade globalization) for our EU 15 sample.^15^ Note that for this variable data are only available from 1970 to 2018. Therefore, the threshold analysis has been constrained to that period. Further information about the components of these variables can be found in Table 14. The source is the KOF Swiss Economic Institute based on the paper of Gygli et al. (2019) and the original contribution of Dreher (2006). The variable to measure economic risk is the 10-year long-term government bond yield differentials between the EA and the United States. The variable is not available for the EU, and the individual variables are not available for the entire period. Nonetheless, the economic risk for the EA can be used as a representative proxy for the economic risk in the EU given that the EA countries are all EU members and represent a large percentage of EU GDP.^16^ Additionally, 12 out of 15 countries in our sample are EA countries. This variable has been obtained from the federal reserve economic database (FRED), provided by the Federal Reserve Bank of St. Louis.^17^ Table 1 summarizes the variables used and data sources.

**Table 1:** Data sources

Variable	Definition	Source
Investment	Gross fixed capital formation over GDP	OECD
Saving	GDP minus total consumption over GDP	OECD
Overall economic openness	KOFEcGI index	KOF Institute
Economic risk	10 year long-term government bond	FRED by the Federal
	differentials between the EA and EEUU	Reserve of St. Louis

### Cross-Section Dependence Test and Order of Integration

We start by applying the Pesaran (2020) cross-sectional dependence test (CD hereafter). The CD test is based on the average of pair-wise correlation coefficients of the OLS residuals from the individual regressions. Pesaran (2020) showed that the test is robust to single or multiple breaks in the slope coefficient and error variances. The CD test is given by:

5 $$\begin{aligned} CD=\sqrt{\frac{2T}{N(N-1}}\left( \sum ^{N-1}_{i=1} \sum ^{N}_{j=i+1}\hat{\rho }_{ij}\right) \end{aligned}$$

Once cross-sectional dependence has been assessed, we test for the order of integration applying Bai and Carrion-i Silvestre (2009) methodology. We have considered the existence of potential and unknown structural changes. This is a non-trivial matter given that unit root tests can lead to misleading conclusions if the presence of structural breaks is not accounted for (see the seminal paper Perron 1989). The panel includes all the individual countries, where we allow for multiple and unknown structural breaks and for cross-country dependence.

Bai and Carrion-i Silvestre (2009) propose a set of panel unit root tests that pool the modified Sarga–-Bhargava (hereafter MSB) tests Sargan and Bhargava (1983) for individual series, both taking into account the possible existence of multiple structural breaks (adapting Bai and Perron (2003) methodology to a panel data framework), and cross-section dependence, modeled as a common factors model as described in Bai and Ng (2004) and Moon and Perron (2004). The common factors may be non-stationary processes, stationary processes, or a combination of both. The number of common factors is estimated using the panel Bayesian criterion information in Bai and Ng (2002). We have used the GAUSS code provided by the authors, allowing for a maximum number of thrree breaks, determined through the Bai and Perron (1998) procedure.^18^

### Cointegration Test

Banerjee and Carrion-i Silvestre (2015) propose a panel cointegration test that allows for structural breaks and cross-sectional dependence under the null of no cointegration. Structural breaks can affect both the deterministic components and the cointegrating vector and they can be treated as known or unknown.

The data generating process (DGP) in structural form as:

6 $$\begin{aligned}&y_{i,t}=D_{i,t}+x^{\prime } _{i,t}\delta _{i,t}+u_{i,t} \end{aligned}$$

7 $$\begin{aligned}&u_{i,t}=F^{\prime }_t\pi _i+e_{i,t} \end{aligned}$$

8 $$\begin{aligned}&(1-L)F_t=C(L)w_t \end{aligned}$$

9 $$\begin{aligned}&(1-\rho _iL)e_{i,t}=H_i(L)\varepsilon _{i,t} \end{aligned}$$

10 $$\begin{aligned}&x_{i,t}=k_i+x_{i,t-1}+G^{\prime }_{t}\zeta _{i} +\Xi _i(L)v_{i,t} \end{aligned}$$

11 $$\begin{aligned}&G_t=\Gamma (L)\bar{\omega }_t \end{aligned}$$

where $i=1,..N, t=1,...,T$, $C(L)=\sum ^{\infty }_{j=0}C_jL^{j}$, $H_i(L)=\sum ^{\infty }_{j=0}h_{i,j}L^{j}$, $\Xi _i(L)=\sum ^{\infty }_{J=0}\Xi _{i,j}L^{j}$ and $\Gamma (L)=\sum ^{\infty }_{j=0}\Gamma _jL^j$. Common factors and factor loadings are estimated using principal components. The optimum number of factors is obtained using the BIC criterion, as recommended by Bai and Ng (2004).

The general functional form for the deterministic term $D_{i,t}$ is

12 $$\begin{aligned} D_{i,t}= \mu _i+\beta _it+\sum \limits ^{m_{i}}_{j=1}\theta _{i,j} DU_{i,j,t}+\sum \limits ^{m_{i}}_{j=1}\gamma _{i,j}DT_{i,j,t} \end{aligned}$$

where $DU_{i,j,t}=1$ and $DT_{i,j,t}=(t-T^{B}_{i,j})$ for $t>T^{b}_{i,j}$ and 0 otherwise. T denoting the timing of the *jth* breaks, $j=1,...,m_i$. The cointegration vector in equation (6) is a function of time:

13 $$\begin{aligned} \delta _{i,t}=\delta _{i,j} for T^{c}_{i,j-1}<t\le T^{c}_{i,j} \end{aligned}$$

where $T^{c}_{i,0}=0$ and $T^{c}_{i,n_{i}+1}=T$, $T^{c}_{i,j}$ and denotes the *jth* time of th break.

The panel cointegration test statistics are obtained based on the sum of the individual ADF:

14 $$\begin{aligned} SADF_j(\lambda )=\sum \limits ^{N}_{i=1}t^{j}_{\tilde{e}^{*}_i} (\lambda _{i}), j=c,\tau , \gamma \end{aligned}$$

The limiting distribution of the statistics is given by:

15 $$\begin{aligned} Z_{j}(\lambda )=\frac{N^{-\frac{1}{2}}SADF_j(\lambda ) -\Theta ^{e}_j(\lambda )\sqrt{N}}{\sqrt{\varPsi _J^e(\lambda )}} \end{aligned}$$

Banerjee and Carrion-i Silvestre (2015) test allows for six possible specifications of the long-run model: from a no-break cointegration vector with an intercept up to structural changes affecting the cointegration vector, the trend and the intercept. The breaks are estimated by minimizing the sum of square residuals as in Bai and Perron (1998).

For comparability, we include Banerjee and Carrion-i Silvestre (2017) panel cointegration test. Banerjee and Carrion-i Silvestre (2017) test also accounts for cross-sectional dependence, but in this case, cross-sectional dependence is approximated by the cross-sectional averages in the spirit of the Pooled Common Correlated Effects (PCCE) approach by Pesaran (2006). Additionally, it is also interesting to compare the results of the cointegration analysis when we do not take into account structural breaks in the data.

Banerjee and Carrion-i Silvestre (2017) test is applied in fourth steps. First, the long-run coefficient is calculated by Pesaran (2006) PCCE. Second, they define the following variable:

16 $$\begin{aligned} \tilde{y}_{i,t}=y_{i,t}-x^{\prime }_{i,t}\hat{\beta }_{PCCE}, \end{aligned}$$

and estimate the following model using OLS:

17 $$\begin{aligned} \tilde{y}_{i,t}=D_{i,t}+\upsilon _{i,t}, \end{aligned}$$

In a third step, the OLS residuals are computed as:

18 $$\begin{aligned} \hat{\upsilon }_{i,t}= \tilde{y}_{i,t}-\hat{D}_{i,t}, \end{aligned}$$

Finally, the null hypothesis of no cointegration is tested applying the Cross-section Augmented Dickey-Fuller cointegration statistic (CADF):

19 $$\begin{aligned} CADF_p=N^{-1}\sum \limits ^{N}_{i=1}t_{\hat{\alpha }_{i,0}}. \end{aligned}$$

### Estimating Long-Run Relationships

According to Pesaran and Smith (1995), there are four procedures to estimate the average effect for large panels: the first is to estimate separate regressions for each group and then average the coefficients over groups, an estimator that is called Mean Group (MG hereafter); the second involves estimating pooled regressions by imposing common slopes and allowing for fixed or random effects; the third involves averaging the data over groups and to estimate aggregate time-series regressions; finally, the fourth involves averaging the data over time and to estimate cross-section regressions on group means. Pesaran and Smith (1995) argued that except for the MG estimator, the pooled and aggregate estimator will yield inconsistent estimates for dynamic models. The reason is that when the regressors are serially correlated, ignoring coefficient heterogeneity causes serial correlation in the disturbance even when $T\rightarrow \infty$.

Pesaran et al. (1999) introduced an intermediate procedure between the MG and the pooled estimator, which is called Pooled Mean Group (PMG hereafter). The PMG constrains the long-run coefficients to be homogeneous but allows the short-run coefficients and error variances to be heterogeneous. The homogeneous long-run coefficients are estimated using the Maximum likelihood (ML) estimator, then the short-run coefficients and the group-specific error correction coefficient are estimated by individual OLS regressions.

Traditional panel methods (including PMG and MG) are built under the assumption of cross-sectional independence across the $i-units$ in the panel. However, this assumption seems to be implausible in many applications.^19^ Cross-sectional dependence may arise due to spillover effects, omitted common effects, or just as a result of the socioeconomic interaction network. To overcome this econometric issue, Pesaran (2006) proposed a new approach to estimate panel data models with a multifactor error structure where the unobserved common factors are allowed to be correlated with the exogenous individual-specific regressors and the factor loadings differ over the cross-section units. The unobserved common factors are eliminated by filtering the individual-specific regressors by the means of cross-section aggregates. This approach is called the Common Correlated Effects estimator^20^ or CCE.

Chudik and Pesaran (2015) extend the CCE to heterogeneous dynamic panel data. This approach is called dynamic common correlated effects (DCCE). They show that the CCE estimator is still valid to deal with the dynamics if two conditions are satisfied: first, a sufficient number of lags of cross-section averages are included, and second, the cross-section averages must be at least as large as the number of unobserved common factors. Chudik and Pesaran (2015) consider the following panel ARDL (1,1) model with a multifactor error structure:

20 $$\begin{aligned} y_{it}=c_{yi}+\phi _iy_{i,t-1}+\beta _{0i}x_{it}+\beta _{1i}x_{i,t-1} +u_{it}, u_{it}=\gamma ^{\prime }_i{\textbf {f}}_t +\varepsilon _{it} \end{aligned}$$

with $i=1,...,N$ and $t=1,..., T_I,$. ${\textbf {f}}_t=(f_{t,1},...,f_{}t,m_f)^\prime$ is the unobserved common factors and $m_f$ the number of factors. Chudik and Pesaran (2015) showed that the common factors in equation (20) can be approximated by the cross-sectional averages of the dependent and independent variables and their $T^{\frac{1}{3}}$ lags:

21 $$\begin{aligned} y_{it}=c_{yi}+\phi _iy_{i,t-1}+\beta _{0i}x_{it}+\beta _{1i}x_{i,t-1} +\sum \limits ^{p_T}_{\ell =0} \delta ^{\prime }_{i\ell }\bar{z}_{t-\ell }+e_{yit} \end{aligned}$$

where $\bar{z}_t=N^{-1}\sum ^{N}_{i=1}z_{it} =(\bar{y}_t,\bar{x}_t,\bar{g}_t)^{\prime }$. $p_T$ is equal to the integer part of $T^\frac{1}{3}$. For this specific case, the mean group estimator of $\pi =E(\pi _i)=(\phi ,\beta ^{\prime }_0, \beta ^{\prime }_1)$ can be estimated as follows:

22 $$\begin{aligned} \hat{\pi }_{MG}=\frac{1}{N}\sum \limits ^{N}_{i=1}\hat{\pi }_i. \end{aligned}$$

#### Error Correction Model and Structural Breaks

The DCCE can be transformed into an error correction model (DCCE-ECM hereafter):

23 $$\begin{aligned} \Delta y_{it}=c_{iy}+\beta _{0i}\Delta x_{it} -\lambda _i (y_{i,t-1}-\gamma _{1i}x_{i,t-1}) +\sum ^{\rho T}_{\ell =0} \delta ^{\prime }_{i\ell } \bar{z}_{t-\ell }+e_{yit} \end{aligned}$$

where $\lambda _i$ and $\gamma _{1i}$ are defined as: $\lambda _i=(1-\phi )$ and $\gamma _{1i}=\frac{\beta _{0i} +\beta _{1i}}{(1-\phi )}$. In Appendix A we show how the DCCE can be transformed into an ECM. One characteristic of the ECM is that there would be a long-run relationship provided that $\lambda _i$ is negative and significant.

In order to test the relationship between domestic investment and domestic saving, the empirical studies commonly used a linear regression model such as:

24 $$\begin{aligned} I_{t}=\alpha +\beta S_{t}+\varepsilon _{t} \end{aligned}$$

The FH relationship to be estimated takes the form:

25 $$\begin{aligned} \Delta I_{it}=c_{iI}+\beta _{0i}\Delta S_{it} -\lambda _i (I_{i,t-1}-\gamma _{1i}S_{i,t-1}) +\sum ^{\rho T}_{\ell =0} \delta ^{\prime }_{i\ell } \bar{z}_{t-\ell }+e_{yit}, \bar{z}=(\bar{I},\bar{S}) \end{aligned}$$

The main advantage of the ECM is that it jointly estimates the short-run dynamics, the long-run equilibrium and the speed of adjustment toward equilibrium. Additionally, to obtain it we can use the MG estimator (CS-MG hereafter) which allows all parameters to be heterogeneous, the PMG estimator (CS-PMG hereafter) allows the short-run dynamics and the speed of adjustment to be heterogeneous while the long-run equilibrium is homogeneous, and finally, the pooled version, that constrains the parameters to be homogeneous. In this paper, we only focus on the CS-MG and the CS-PMG estimations.^21^ The Hausman test is used to choose between the CS-MG and CS-PMG estimators.^22^

Last, we extend the DCCE-ECM estimator by allowing structural breaks as in Camarero et al. (2016). Based on the break dates and the models obtained from the Banerjee and Carrion-i Silvestre (2015) cointegration approach, we include the break in our DCCE-ECM by filtering the variables by the break.^23^ In this paper, we only include one homogeneous break, since the heterogeneous breaks analysis has already been covered in Camarero et al. (2021).

### Threshold Analysis Applying CS-DL

Chudik et al. (2016) develop a cross-sectionally augmented distributed lags (CS-DL hereafter) approach to estimate the long-run effects in large dynamic heterogeneous panel data with cross-sectional dependence. The CS-DL estimator is robust to misspecification of dynamics and error serial correlation. Moreover, the CS-DL approach is superior to ARDL when T is not too large. The CS-DL estimator is based on the following auxiliary regression:

26 $$\begin{aligned} y_{it}=c_{yi}+\theta ^\prime _ix_{it} +\sum \limits ^{p-1}_{\ell =0}\delta _{i\ell } \Delta x_{i,t-\ell }+\sum \limits ^{p_{\bar{y}}}_{\ell =0} \omega _{y,i\ell }\bar{y}_{t-\ell } +\sum \limits ^{p_{\bar{x}}}_{\ell =0}\omega ^\prime _{x,i\ell } \bar{x}_{t-\ell }+e_{it} \end{aligned}$$

where $\bar{x}_t=N^{-1}\sum ^{N}_{i=1}x_{it}$ and $\bar{y}_t=N^{-1}\sum ^{N}_{i=1}y_{it}$, $p_{\bar{x}}$ is set equal to the integer part of $T^{\frac{1}{3}}$, $p=p_{\bar{x}}$ and $p_{\bar{y}}$ is set to 0.

We apply the methodology proposed by Chudik et al. (2016) to estimate the threshold model.^24^ They investigate whether the debt-growth relationship varies with the level of indebtedness. To do this, they develop tests for threshold effects using the CS-DL estimator.^25^ Chudik et al. (2017) choose the level of indebtedness as the threshold variable, that is also included in the model.^26^ In our case, the objective is to estimate the saving retention coefficient at different levels of risk and openness. The threshold variables (economic risk and financial plus trade openness) do not enter the estimated equation but they restrict it depending on whether they are above or below the threshold. Our specific threshold model takes the following form:

27 $$\begin{aligned} I_{it\tau _{+}}&=c_{Ii\tau _{+}}+\theta ^\prime _iS_{it\tau _{+}} +\sum \limits ^{p-1}_{\ell =0}\delta _{i\ell \tau _{+}} \Delta S_{i,t-\ell ,\tau _{+}}+\sum \limits ^{p_{\bar{I}}}_{\ell =0} \omega _{I,i\ell \tau _{+}}\bar{I}_{t-\ell ,\tau _{+}}\nonumber \\&\quad +\sum \limits ^{p_{\bar{S}}}_{\ell =0}\omega ^\prime _{S,i\ell \tau _{+}} \bar{S}_{t-\ell ,\tau _{+}}+e_{it\tau _{+}} \end{aligned}$$

28 $$\begin{aligned} I_{it\tau _{-}}&=c_{Ii\tau _{-}}+\theta ^\prime _i S_{it\tau _{-}}+\sum \limits ^{p-1}_{\ell =0}\delta _{i\ell \tau _{-}} \Delta S_{i,t-\ell ,\tau _{-}}+\sum \limits ^{p_{\bar{I}}}_{\ell =0} \omega _{I,i\ell \tau _{-}}\bar{I}_{t-\ell ,\tau _{-}}\nonumber \\&\quad +\sum \limits ^{p_{\bar{S}}}_{\ell =0}\omega ^\prime _{S,i\ell \tau _{-}} \bar{S}_{t-\ell ,\tau _{-}}+e_{it\tau _{-}} \end{aligned}$$

When risk and openness are larger than the threshold level (represented by subindex $\tau _{+}$), Eq. (27) is estimated. In contrast, when risk and openness are lower than the threshold level (represented by subindex $\tau _{-}$) Eq. (28) is estimated.

The thresholds have been chosen based on two factors: first, the thresholds should define the limit between low and high economic risk and a low and high degree of openness; second, we set a minimum of periods to be estimated of $T>25$.^27^ Hence, the thresholds are defined as follows: for risk, we set a low degree of economic risk for a spread lower than 70 basis points (0.7 in our data),^28^ whereas risk is high when the spread is over 70. For the openness index, we set the threshold at 70$\%$, so that over this level openness is considered to be high.

## Results

### Dependence and Order of Integration

We present in the first part of Table 2 the results of the Pesaran (2020) CD test on the individual variables. We have included both, Case 2 (model with constant) and 3 (model with constant and trend) since there is no clear pattern in the series and we did not want to restrict the analysis to one model. The results of the CD test on saving and investment show strong evidence against the null of cross-sectional independence. In the second part of Table 2 we present the CD test applied to the saving-investment regression (cases 2 and 3). In line with the individual time series analysis, we find strong evidence against the null hypothesis of cross-sectional independence. From these results, we conclude that cross-sectional dependence should be taken into account.

**Table 2:** Pesaran (2020) cross-sectional dependence test (CD)

1.1 CD on the time series				
$\rho$	Model 2: intercept		Model 3: intercept and trend	
	Investment	Saving	Investment	Saving
0	$$15.48^{***}$$	$$24.79^{***}$$	$$15.66^{***}$$	$$22.24^{***}$$
1	$$11.84^{***}$$	$$24.59^{***}$$	$$12.28^{***}$$	$$21.92^{***}$$
2	$$11.84^{***}$$	$$23.18^{***}$$	$$12.38^{***}$$	$$21.21^{***}$$
3	$$11.86^{***}$$	$$23.35^{***}$$	$$12.24^{***}$$	$$21.72^{***}$$

1.2 CD on the FH regression				
WCD	Model 2: intercept		Model 3: intercept and trend	
$$CD-statistic$$	$$10.60^{***}$$		$$10.76^{***}$$	

The WCD test is carried out at the 2-sided nominal significant level. ** and *** denote the rejection of weak cross-sectional dependence at the $5\%$ and $1\%$ levels, respectively

For the CD performed on the time series, $\rho$ is the autoregressive order following an AR($\rho$)

CD test on the FH regression is performed on the Pesaran et al. (1999) Pooled Mean Group

Next, in Table 3 we present the panel unit root tests that account for cross-section dependence. When allowing for common factors and structural breaks, the results support the non-stationarity of the two variables, saving and investment.^29^ Moreover, we also find strong evidence of multiple structural breaks affecting most of the variables analyzed, differing in number and position for individual countries, as shown in Table 4. Thus, we can conclude that the variables in Table 3 are I(1) with structural breaks.

**Table 3:** Bai and Carrion-i Silvestre (2009) Panel Unit Root Test with common factors and structural breaks Period 1970–2019. Model 2 (change in the constant and trend)

Variables	Approach	*Z*	$P_{m}$	*P*	$Z^*$	$P^*_m$	$P^*$	*T*	*N*	*m*	*fr*
Investment	BIC	0.62	$-$1.02	22.12	13.11***	$-$1.15	21.13	50	15	3	46
	S	0.62	$-$1.02	22.12	13.11***	$-$1.15	21.13	50	15	3	46
Saving	BIC	$-$1.38	0.77	35.99	4.55	0.49	33.80	50	15	3	46
	S	3.49***	$-$1.75	16.47	7.59	$-$1.69	16.90	50	15	3	46

*Z*, *P* and $P_m$ denote the test statistics proposed by Bai and Carrion-i Silvestre (2009). *Z* and $P_m$ follow a standard normal distribution and their $1\%$, $5\%$ and $10\%$ critical values are 2.326, 1.645 and 1.282, respectively. *P* follows a Chi-squared distribution with N x breaks (*m*) + 1 degrees of freedom (*fr*) and critical values 69.96, 61.66 and 57.51, at $1\%$, $5\%$ and $10\%$, respectively. The number of common factors is chosen using the panel Bayesian information criterion proposed by Bai and Ng (2002). $Z ^*$, $P^{*}$ and $P^*_m$ refer to the corresponding statistics obtained using the p-values of the simplified MSB statistics. One, two and three asterisks denote the rejection of the null hypothesis of a unit root at $10\%$, $5\%$ and $1\%$ significance levels, respectively, when the statistic is greater than the upper level

BIC and S denote the BIC and Sequential procedure used to select the number of breaks according to Bai and Perron (2003)

**Table 4:** Bai and Carrion-i Silvestre (2009). Structural breaks

Country	Investment		Saving	
	BIC	S	BIC	S
Belgium	1977	1977	-	1979
Denmark	-	-	-	2006
France	1997	1997	-	-
Germany	1977	1977	-	-
Greece	-	-	-	2007
Ireland	2011	2011	-	-
Netherlands	-	-	1996	-
	-	-	2003	-
Spain	-	-	2010	-

BIC and S denote the BIC and Sequential procedure used to select the number of breaks according to Bai and Perron (2003)

### Cointegration Results and Structural Breaks

Once we have assessed the order of integration of the variables, we move to the analysis of cointegration. The results of the Banerjee and Carrion-i Silvestre (2015) test for one and two heterogeneous breaks are presented in tables 5 and 6, respectively. When we consider just one heterogeneous break, we find that the null of no cointegration is rejected for model 3 (chosen according to the AIC and BIC criteria). The first salient feature is that the location of the break coincides with the 2008–10 financial crisis for the majority of countries.^30^ With some exceptions, the other breaks are found either at the end of the 70 s, when the European Monetary System was adopted(Belgium, Ireland and Luxembourg^31^), the end of the 90 s for Portugal, coinciding with the inception of the Euro or, in the case of Finland, in the early 90 s, capturing the Finnish crisis after the collapse of the Soviet Union.^32^ In the case of two heterogeneous breaks (table 6), we cannot reject the null of no cointegration in the chosen model (model 3). However, we can reject the null for model 4, which allows for breaks in the cointegration vector. The first break in model 4 is found at the beginning of the 80 s or late 70 s with the EMS creation (1979), and later, during the 80 s, parallel to the financial liberalization measures of the Single Market project. However, in some cases, such as France and Italy, the first break is found in the 90 s, at the Maastricht treaty signature and the EMS crisis. Regarding the second break, we can split the analysis into two groups: core countries and periphery.^33^ For the first group, the break occur at the Euro inception (Austria, Belgium, Germany) and the Maastricht treaty/Single Market (Finland,^34^ Luxembourg and Sweden). UK’s break is found after the Pound left the EMS, whereas the second break for Denmark is found in 1987.^35^ Regarding the peripheral countries, the 2007–2008 financial crisis and the sudden stop of capital inflows provoked large external imbalances that led to the sovereign debt crisis. Therefore, the second break for this group is found around the financial and sovereign crisis.^36^

**Table 5:** Banerjee and Carrion-i Silvestre (2015) panel cointegration test with 1 heterogeneous break

1 break	Model 1	Model 2	Model 3	Model 4	Model 5
Austria	–	–	2010	1981	1981
Belgium	–	–	1980	1980	1980
Denmark	–	–	2008	1980	1980
Finland	–	–	1990	1992	1992
France	–	–	2008	1996	1996
Germany	–	–	2009	2001	2001
Greece	–	–	2005	1977	1977
Ireland	–	–	1981	1980	1979
Italy	–	–	2008	1992	1992
Luxembourg	–	–	1983	1985	1985
Netherlands	–	–	2006	1980	1980
Portugal	–	–	2002	1983	1983
Spain	–	–	2008	2008	2008
Sweden	–	–	2008	1992	1992
UK	–	–	2008	1980	1980
$$Z^*_J$$	$$-2.43^{***}$$	$$-$$0.55	$$-3.43^{***}$$	$$-$$2.14	$$-$$1.03
*AIC*	$-$7.85	−8.80	**−9.12**	−8.36	−8.95
*BIC*	$-$7.66	−8.53	**−8.75**	−7.99	−8.48

Bold values indicate the preferred model based on the information criteria. Model 1 is a stable cointegration model with constant; model 2 is a stable cointegration model that includes a trend; model 3 includes a constant and a trend allowing for changes in the constant; model 4 includes no trend and the constant and the cointegration vector may have structural changes; model 5 includes a stable trend and the constant and the cointegration vector may have structural changes

Following Banerjee and Carrion-i Silvestre (2015), Model 3 (with breaks in the trend) and model 6 cannot be performed for the cases of heterogeneous unknown breaks due to the fact that the trend cannot have heterogeneous breaks

** and *** denote the rejection of the null of no cointegration at the 5% and 1% levels, respectively. Critical values for the $Z^*_j$ statistic are found in Banerjee and Carrion-i Silvestre (2015) table III

$Z^*_t$ statistic is estimated with a maximum of six common factors and up to the twelfth autoregressive order for the $AR(\rho )$

**Table 6:** Banerjee and Carrion-i Silvestre (2015) panel cointegration test with 2 heterogeneous breaks

2 breaks	Model 1	Model 2	Model 3	Model 4	Model 5
Austria	–	–	1981	1981	1981
	–	–	2008	2001	2001
Belgium	–	-	1980	1980	1980
	–	-	1987	2001	2001
Denmark	–	–	1980	1980	1980
	–	-	2008	1987	1987
Finland	–	–	1990	1977	1977
	–	–	2008	1992	1992
France	–	–	1992	1996	1996
	–	–	2008	2006	2006
Germany	–	–	1988	1980	1980
	–	–	2009	2001	2001
Greece	–	–	2002	1983	1983
	–	–	2010	2008	2008
Ireland	–	–	1981	1980	1979
	–	–	2004	2009	2009
Italy	–	–	1996	1992	1992
	–	–	2008	2011	2011
Luxembourg	–	–	1985	1985	1985
	–	–	1993	1992	1992
Netherlands	–	–	1987	1980	1980
	–	–	2006	2009	2006
Portugal	–	–	1986	1983	1983
	–	–	2002	2011	2011
Spain	–	–	1986	1977	1977
	–	–	2008	2008	2008
Sweden	–	–	1990	1977	1977
	–	–	2008	1992	1992
UK	–	–	1979	1980	1980
	–	–	2008	1996	1996
$$Z^*_J$$	$$-2.43^{***}$$	$$-$$0.55	$$-$$0.94	$$-2.60^{**}$$	$$-$$0.97
*AIC*	$-$7.86	$-$8.80	**−9.10**	−8.13	−9.00
*BIC*	$-$7.67	$-$8.53	**−8.64**	−7.57	−8.35

Bold values indicate the preferred model based on the information criteria. Model 1 is a stable cointegration model with constant; model 2 is a stable cointegration model that includes a trend; model 3 includes a constant and a trend allowing for changes in the constant; model 4 includes no trend and the constant and the cointegration vector may have structural changes; model 5 includes a stable trend and the constant and the cointegration vector may have structural changes

Following Banerjee and Carrion-i Silvestre (2015), Model 3 (with breaks in the trend) and model 6 cannot be performed for the cases of heterogeneous unknown breaks due to the fact that the trend cannot have heterogeneous breaks

*, ** and *** denote the rejection of the null of no cointegration at the 10% 5% and 1% levels, respectively. Critical values for the $Z^*_j$ statistic are found in Banerjee and Carrion-i Silvestre (2015) table III

$Z^*_t$ statistic is estimated with a maximum of six common factors and up to the twelfth autoregressive order for the $AR(\rho )$

Table 7 shows the Banerjee and Carrion-i Silvestre (2015) test for one homogeneous break. In this case, we can reject the null of no cointegration for all the models, with the exception of model 1. According to the AIC and BIC criteria, the best model is model 3, which allows for a break in the intercept and the trend. In this model, the break is found in 2001, at the inception of the euro, when the exchange rate risk was significantly reduced after a period of rapid financial integration. Therefore, if we restrict the endogenous selection procedure to find a single common break, the creation of the euro is the most important factor affecting the domestic investment and saving relationship for our sample.

**Table 7:** Banerjee and Carrion-i Silvestre (2015) panel cointegration test with 1 homogeneous break

1 break	Model 1	Model 2	Model 3	Model 4	Model 5	Model 6
$$Z^*_j$$	$$-$$0.48	$$-3.24^{***}$$	$$-5.09^{***}$$	$$-3.20^{***}$$	$$-4.80^{***}$$	$$-7.05^{***}$$
*AIC*	−7.98	−9.05	**−9.74**	−7.70	−8.91	−9.69
*BIC*	−7.80	−8.77	**−9.28**	−7.33	−8.45	−9.13
*Break*	-	-	2001	1983	1991	2001

Bold values indicate the preferred model based on the information criteria. Model 1 is a stable cointegration model with constant; model 2 is a stable cointegration model that includes a trend; model 3 includes a constant and a trend allowing for changes in both; model 4 includes no trend and the constant and the cointegration vector may have structural changes; model 5 includes a stable trend and the constant and the cointegration vector may have structural changes; model 6 allows for breaks in the constant, the trend and the cointegration vector

** and *** denote the rejection of the null of no cointegration at the 5% and 1% levels, respectively. Critical values for the $Z^*_j$ statistic are found in Banerjee and Carrion-i Silvestre (2015) table III

$Z^*_t$ statistic is estimated with a maximum of six common factors and up to the twelfth autoregressive order for the $AR(\rho )$

For comparability, we report in table 8 the results from the Banerjee and Carrion-i Silvestre (2017) panel cointegration test, which shows no evidence of cointegration. Two potential reasons may explain this result: first, the presence of structural breaks unaccounted for; second, the role of dynamics in the investment-saving relationship. The CCE estimator does not have a good dynamic performance. Therefore, we use the DCCE estimator, which is a more suitable alternative.

**Table 8:** Banerjee and Carrion-i Silvestre (2017) panel cointegration test using Pesaran’s PCCE estimator

$\rho$	Model 1: intercept		Model 2: intercept and trend	
	$r=1$	$r=2$	$r=1$	$r=2$
0	$-2.04$	$-2.21$	$-2.26$	$-2.12$
1	$-2.25$	$-2.49$	$-2.48$	$-2.37$
2	$-1.91$	$-2.06$	$-2.18$	$-2.03$

*r* refers to the number of common factors and $\rho$ is the order of the autoregressive correction for the panel cointegration CCE statistic (CADF)

Following Banerjee and Carrion-i Silvestre (2017), the number of common factor (r) is assumed to be the same as the k variables (rank condition is met with inequality) or k+1 variables (rank condition is met with equality) in the long-run specification of the Pooled Common Correlated Effects (PCCE). In our case, r=1 and r=2, respectively

** and *** denote the rejection of the null of no cointegration at the 5% and 1% levels, respectively. Critical values are found in Banerjee and Carrion-i Silvestre (2017) table 1, 2, 3 and 4

### Implications in Terms of Capital Mobility and the FH Puzzle

Once we have assessed the order of integration of the variables, as well as the existence of cointegration between domestic investment and saving, the next step is to analyze the FH puzzle. We start with the traditional FH regression with no breaks (model 1).^37^ The regression with no structural breaks is included for comparability reasons since it is not the preferred model according to the AIC and BIC criteria. Concerning the regression with breaks, we report first the results with one homogeneous break. Keeping in mind the original interpretation of the FH regression, we can adapt it to our ECM model. When we obtain a parameter equal to 1 or 0 for the long-run and short-run parameters, there is capital immobility in the former case and perfect capital mobility in the latter. When the parameter is between 0 and 1, the degree of capital mobility is moderate to high. Negative values will be also interpreted as perfect capital mobility. Regarding the error correction term, it measures the speed of adjustment towards equilibrium. Therefore, if the speed is slow it will be taken as evidence in favor of capital mobility since the gap between investment and saving needs to be filled by international capital flows or investing abroad if $S>I$ (countries with current account surpluses).

Regarding the regression without breaks, we present the results for the CS-PMG estimations in table 9 (left side). The Hausman test of homogeneous long-run coefficients cannot be rejected, therefore, we will interpret the estimates from the CS-PMG model since they are more efficient than the CS-MG estimator with long-run heterogeneous coefficients. We report the country analysis in Table 10. Our main findings are, first, that the coefficient in the model without trend is large (1.27 but statistically equal to one) and significant. This evidence points in favor of strong capital immobility. However, this conclusion should be qualified if we take into account the error correction model since there is no short-run impact of domestic saving in domestic investment.^38^ Additionally, the error correction parameters show a slow return to the equilibrium (12 years on average), which would point to capital mobility. The error correction parameters are significant and negative for all the countries with a long-run relationship. Second, the peripheral countries (except for Ireland) have slower returns to the equilibrium than the core countries. This persistence in the investment-saving adjustment is in line with the large external imbalances observed from the inception of the euro up to the Great Recession.

**Table 9:** Panel Chudik and Pesaran (2015) DCCE-ECM Panel results

Parameter	1970–2019	
	Model 1 (traditional FH)	Model 3 (with breaks)
	DCCE(CS-PMG)	DCCE (CS-PMG)
LR parameter	1.27***	0.34***
(s.e.)	(0.22)	(0.05)
EC term	− 0.14***	− 0.18***
(s.e.)	(0.02)	(0.05)
SR parameter	− 0.13**	− 0.04
(s.e.)	(0.05)	(0.07)
Hausman test	2.45	1.36
(*p*-value)	(0.12)	(0.24)

*, ** and ** denote statistical significance at the $10\%$, $5\%$ and $1\%$ levels. Following Chudik and Pesaran (2015), the cross-sectional averages and $T^{1/3}$ lags are included to account for cross-sectional dependence. Number in brackets are standard errors

LR, EC and SR stand for long-run, error correction and short-run, respectively

DCCE(CS-PMG) refers to the DCCE estimated by the PMG

For the Hausman test, *, ** and ** denote the rejection of the null of long-run parameter homogeneity at the $10\%$, $5\%$ and $1\%$ levels

**Table 10:** Chudik and Pesaran (2015) DCCE-ECM (CS-PMG) country analysis (no break model with constant)

EU 15	Model with constant and no breaks 1970–2019			
Country	LR parameter	EC term	SR parameter	Years to return
Austria	1.27***	$-$0.11***	$-$0.01	9
	(0.22)	(0.01)	(0.04)	
Belgium	1.27***	$-$0.13***	0.42***	8
	(0.22)	(0.01)	(0.02)	
Denmark	1.27***	$-$0.08***	$-$0.01	11
	(0.22)	(0.01)	(0.03)	
Finland	1.27***	$-$0.22***	$-$0.13***	5
	(0.22)	(0.01)	(0.02)	
France	1.27***	$-$0.04***	0.06*	25
	(0.22)	(0.02)	(0.02)	
Germany	1.27***	$-$0.21***	$-$0.30**	5
	(0.22)	(0.01)	(0.01)	
Greece	1.27***	$-$0.08***	0.03	13
	(0.22)	(0.01)	(0.04)	
Ireland	1.27***	$-$0.29***	$-$0.30***	3
	(0.22)	(0.01)	(0.04)	
Italy	1.27***	$-$0.05***	$-$0.27***	20
	(0.22)	(0.01)	(0.01)	
Luxembourg	1.27***	$-$0.26***	$-$0.38***	4
	(0.22)	(0.01)	(0.02)	
Netherlands	1.27***	$-$0.23***	$-$0.39***	4
	(0.22)	(0.01)	(0.06)	
Portugal	1.27***	$-$0.03***	$-$0.12***	33
	(0.22)	(0.01)	(0.01)	
Spain	1.27***	$-$0.04***	$-$0.29***	25
	(0.22)	(0.01)	(0.03)	
Sweden	1.27***	$-$0.26***	$-$0.17***	4
	(0.22)	(0.01)	(0.02)	
United Kingdom	1.27***	$-$0.10***	$-$0.17***	11
	(0.22)	(0.01)	(0.01)	
Average years to return				12
Model selection criteria	BIC (1,1)			

*, ** and ** denote statistical significance at the $10\%$, $5\%$ and $1\%$ levels. Following Chudik and Pesaran (2015), the cross-sectional averages and $T^{1/3}$ lags are included to account for cross-sectional dependence. Number in brackets are standard errors

LR, EC and SR stand for long-run, error correction and short-run, respectively

The above analysis, where no breaks are allowed in the model, stresses the relevance of correctly including structural breaks in the study. Interestingly, when breaks are not considered, the results show a shallow level of economic integration and capital mobility in the long run. Therefore, not taking into account breaks in the analysis may underestimate capital mobility.^39^ Non-linearity can be introduced by including structural breaks or by performing a threshold analysis. This is the goal of the analysis below.

In Tables 9 (right side) and 11, we report the panel and country-by-country results for model 3 (break model). Based on the results of the Hausman test in table 9, we cannot reject the null hypothesis of homogeneity of the long-run parameters; hence, the efficient estimators are those obtained in the CS-PMG case.

**Table 11:** Chudik and Pesaran (2015) DCCE-ECM (CS-PMG) country analysis with one break (model 3)

EU 15	1970–2019			
Country	LR parameter	EC term	SR parameter	Years to return
Austria	0.34***	$-$0.12***	$-$0.09	8
	(0.05)	(0.01)	(0.05)	
Belgium	0.34***	$-$0.12***	0.49***	8
	(0.05)	(0.01)	(0.01)	
Denmark	0.34***	$-$0.08***	0.05	12
	(0.05)	(0.01)	(0.04)	
Finland	0.34***	$-$0.17***	0.12***	6
	(0.05)	(0.01)	(0.02)	
France	0.34***	$-$0.03***	0.19**	30
	(0.05)	(0.01)	(0.02)	
Germany	0.34***	$-$0.19***	$-$0.14***	5
	(0.05)	(0.01)	(0.01)	
Greece	0.34***	$-$0.11***	0.40***	9
	(0.05)	(0.01)	(0.04)	
Ireland	0.34***	$-$0.11***	0.01	9
	(0.05)	(0.01)	(0.04)	
Italy	0.34***	$-$0.11***	$-$0.29***	9
	(0.05)	(0.01)	(0.01)	
Luxembourg	0.34***	$-$0.52***	$-$0.39***	2
	(0.05)	(0.01)	(0.02)	
Netherlands	0.34***	$-$0.77***	$-$0.43***	2
	(0.05)	(0.02)	(0.04)	
Portugal	0.34***	$-$0.05***	$-$0.09***	20
	(0.05)	(0.01)	(0.01)	
Spain	0.34***	$-$0.08***	$-$0.36***	13
	(0.05)	(0.01)	(0.03)	
Sweden	0.34***	$-$0.22***	0.06**	5
	(0.05)	(0.01)	(0.02)	
United Kingdom	0.34***	$-$0.14***	$-$0.17***	7
	(0.05)	(0.01)	(0.01)	
Average years to return				10
Model selection criteria	BIC (1,1)			

*, ** and ** denote statistical significance at the $10\%$, $5\%$ and $1\%$ levels. Following Chudik and Pesaran (2015), the cross-sectional averages and $T^{1/3}$ lags are included to account for cross-sectional dependence. Number in brackets are standard errors

LR, EC and SR stand for long-run, error correction and short-run, respectively

Table model 3 (Table 11) shows the results in our estimation. The break coincides with the inception of the euro. In this case, the value of the long-run retention parameter drastically drops to 0.34. This result shows that capital mobility is relatively high, although it is not perfect (following the definition of Feldstein and Horioka (1980)). According to Ford and Horioka (2017), a zero coefficient in the saving retention parameter means perfect integration in both goods and financial markets. Therefore, our results suggest that economic integration is relatively high for our EU sample, although it is still incomplete. Regarding capital mobility in the short-run, it is generally high, with some exceptions; Belgium and Greece, for which we observe a moderate degree of capital mobility. For Finland and France, despite observing an impact on domestic saving on domestic investment in the short-run, we can conclude that capital mobility is relatively high (although not perfect). The error correction parameters are significant and negative for all the countries. The average speed of adjustment for the panel is ten years. Again, some patterns in the speed of adjustment become apparent: for the northern countries, the adjustment is moderately quick, taking six years on average. In contrast, the peripheral countries show a slower speed of adjustment, with an average of twelve years. However, the speed of adjustment varies across them, being more sluggish for Spain and Portugal. Compared with the no-break case, Ireland is a peripheral country. This analysis, in general, concurs with the observed patterns and the persistence of the imbalances, as a slow return to the equilibrium may induce those countries to keep borrowing from abroad, enlarging their external imbalance.

We can compare our results with other papers that test the FH puzzle using a similar sample. To the best of our knowledge, only three papers have studied the same country group. Camarero et al. (2020) found that the saving-investment coefficient has been dropping over time, implying an increase in capital mobility. The authors also highlight the heterogeneity of FH relationship, even among highly integrated economies. We find similar broad results, although the econometric techniques are different. Drakos et al. (2017, 2018) using the panel cointegration methodology found moderate capital mobility. However, as we have shown, the degree of capital mobility can be underestimated if structural breaks are not taken into account.

### Threshold Results

**Fig. 1:**
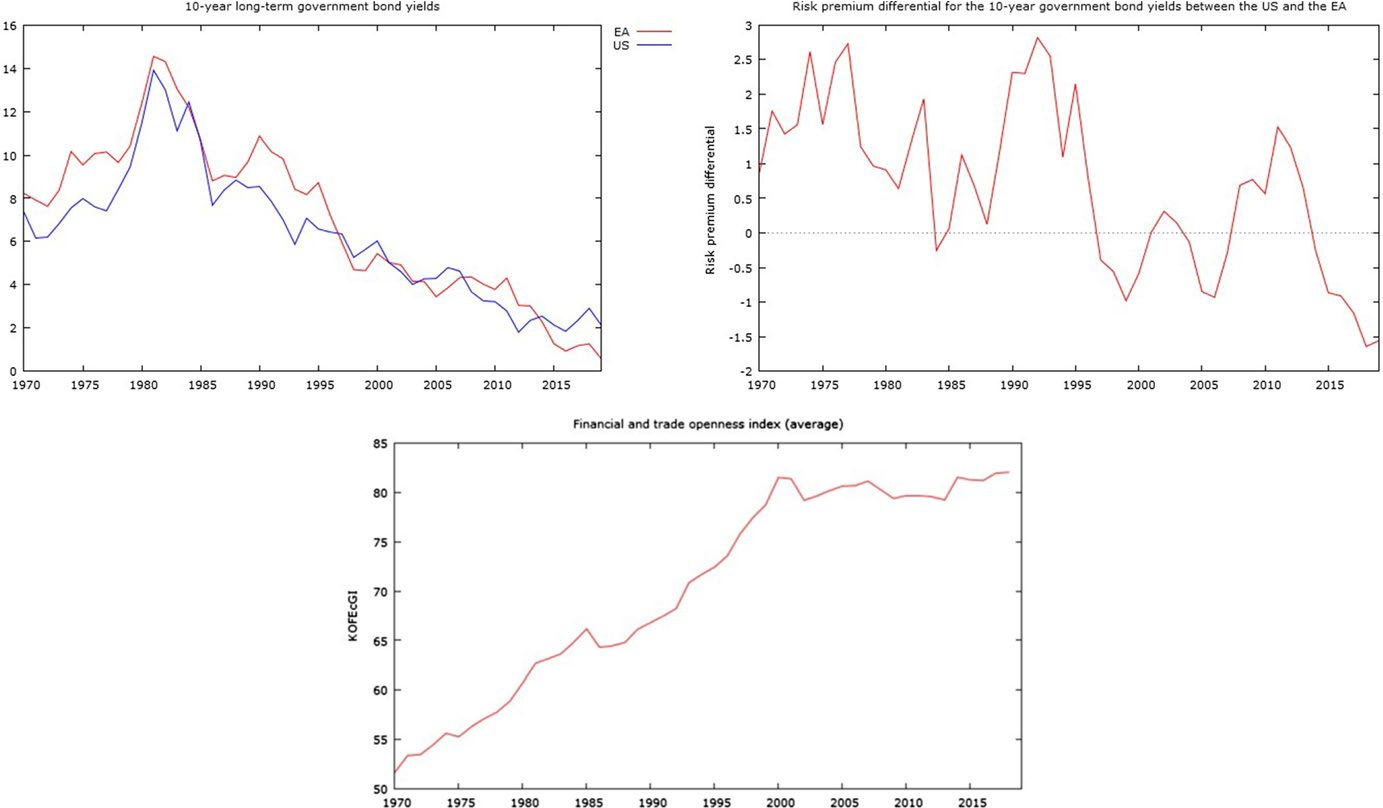
Risk, trade and financial openness index figures

Finally, in the last step of our analysis, we estimate the saving retention coefficient at different levels of risk and openness. The objective is to assess if capital mobility and economic integration may change when a certain level of openness and risk is reached. Prior to the regression results, we briefly describe the patterns observed in the graphs of Fig. 1. The first two graphs show the 10-year government bond yields for the EA and the US (left-hand-side graph). We can observe a clear downward trend since 1990. In the right-hand side graph we show the risk premium, that is, the differential between the US and the EA 10-year government bond.^40^ The risk premium differential displays some remarkable features: First, the differential has been higher during the periods of crises in the EA countries (the EMS crisis in the early 90 s and the sovereign crisis). In contrast, the euro had an unprecedented low-risk period up to the financial crisis. In addition, the graph shows how the EMS has contributed to financial stability (until the early 90 s). The third graph shows the overall openness index for the period 1970–2018. The upward trend observed until the euro coincides with the financial integration policies that started in the 70 s. After the launching of the euro, openness has remained high and stable.

We now apply the threshold methodology to our data, looking for the potential impact of risk and openness on capital mobility. The previous results confirm the importance of some economic milestones behind the reduction of the saving retention coefficient. However, these nonlinearities can also be captured using variables that may explain the changes between regimes. In Tables 12 and 13 we show the results. In the case of risk (Table 12), when it is perceived as high, we observe a significant long-run parameter that reaches 0.71 (capital immobility). As we found in our previous analysis, high risk was present during the EMS crisis in the early 90 s and, more recently, during the sovereign debt crisis. In contrast, we found that the saving retention coefficient drops to zero when the risk is lower than the threshold (low-risk stage).^41^ These results show that capital mobility tends to decrease in periods of risk. However, capital mobility and economic integration increase in periods of low risk. We turn now to the threshold analysis for the openness variable. When we use the financial and trade openness index (see Table 13), if openness is low, we find a moderate level of capital mobility. However, for high values of the index, the FH coefficient turns out to be statistically not different from zero. In line with Ford and Horioka (2017), this result means that when openness is high, the saving retention coefficient provides evidence in favor of integrated capital and goods markets for our EU-15 group. It should be noted that the index reached its highest value after 1994,^42^ as its level remains a bit over $70\%$. According to this, perfect capital mobility was achieved even before the euro’s inception, with the EU’s removal of trade and capital controls.

**Table 12:** Chudik et al. (2016) CS-DL threshold model with risk variable

Parameter	1970–2019		
	Spread < 0.7	Spread < 1	Spread > 0.7
Long-run coefficient	0.02	0.16	0.71**
(standard error)	(0.33)	(0.17)	(0.31)
Constant	0.02	0.00	$-$0.00
(standard error)	(0.00)	(0.00)	(0.00)
T	25	30	25
N	15	15	15
NxT	375	450	375

*, ** and ** denote statistical significance at the $10\%$, $5\%$ and $1\%$ levels. Following Chudik and Pesaran (2015), the cross-sectional averages and $T^{1/3}$ lags are included to account for cross-sectional dependence. According to the sample size, 2 distributed lags of saving have been included

Number in brackets are standard errors

**Table 13:** Chudik et al. (2016) CS-DL threshold model with trade and financial openness index

Parameter	1970–2018	
	Index lower than 70$\%$	Index larger than 70$\%$
Long-run coefficient	0.58***	0.04
(standard error)	(0.17)	(0.10)
Constant	0.00	0.00
(standard error)	(0.00)	(0.00)
T	24	25
N	15	15
NxT	360	375

*, ** and ** denote statistical significance at the $10\%$, $5\%$ and $1\%$ levels. According to the sample size, the cross-sectional averages, $T^{1/3}$ lags are included to acount for cross-sectional dependence. According to the sample size, 2 distributed lags of saving have been included

Number in brackets are standard errors

Both DCCE and threshold analysis provide evidence in favor of capital and economic integration; however, the latter gives more precise results. Note that both methods are complementary: while the DCCE approach estimates the whole period (1970–2019), including a structural break in 2001 (euro effect), the threshold provides information to distinguish between regimes with different degrees of risk and openness.

## Conclusions

This paper studies the evolution of capital mobility in the EU-15 during the period 1970–2019 using annual data. We adopt the quantity approach, unlike most of the earlier literature, that focuses on the price approach. Our perspective can be especially suited to assess the evolution of capital mobility in the EU, where external imbalances have been a recurring problem for financial stability, especially during the last two decades, where they were persistently growing up to the 2008 financial crisis. From an econometric standpoint, we base our analysis on recent panel data techniques that exploit the cross-section and time information involved in large T-panels considering key statistical issues usually neglected in previous literature: cross-sectional dependence, structural breaks, endogeneity and dynamics.

The main contributions of our analysis are: first, we provide a complete assessment of capital mobility in the EU-15, studying not only the long-run but also providing evidence about the short-run dynamics towards equilibrium. Second, we have an appraisal on how capital mobility evolves when structural breaks are accounted for. Third, we provide empirical evidence of the Ford and Horioka (2017)’s hypothesis, according to which both financial and goods integrated markets are necessary conditions to obtain a zero coefficient in the saving retention parameter. Fourth, we analyze whether large and persistent external imbalances can be explained by the long-run relationship between domestic saving and investment; finally, we obtain an assessment of the impact of risk and openness on capital mobility.

Regarding our first three questions, when we do not account for structural breaks, our results confirm capital immobility and a very low level of economic integration in the long-run. However, capital mobility is high in the short-run. When we include multiple heterogeneous structural breaks in our analysis, we observe that the long-run domestic saving-investment relationship has been affected by the creation of the EMS, as well as by the EMS crisis in the early 90 s, the Maastricht Treaty and the financial and sovereign crises. But we find the homogeneous common break at the inception of the euro. When we incorporate the euro launching as a break in our estimation, the long-run saving retention coefficient drastically decreases, showing an improvement in capital mobility. Thus, according to Ford and Horioka (2017), the saving retention parameter obtained shows that capital mobility is high, however, the results also suggest that both, financial and real integration are incomplete. This finding is a salient feature of this paper in comparison with earlier studies and highlights the importance of accounting for structural breaks, stressing the key role played by the euro as a booster for both financial and real integration. As our methodology assesses long-run behavior, the impact of the financial and sovereign crisis cannot be isolated. Nonetheless, our threshold analysis is able to capture the financial and debt crises, characterized as a high risk period, and shows how the I-S relationship changes when risk and openness are high.

Concerning the speed of adjustment towards the long-run equilibrium, while the core countries tend to return to the equilibrium rather quickly (six years), peripheral countries return at a lower speed (twelve years). This very long path towards equilibrium, together with the savings glut generated in the core countries and the removal of capital controls may explain the large and persistent external imbalances observed within the EU-15.

Finally, we give a glimpse at some of the factors that may affect capital mobility: risk and openness. Using threshold models, we find that capital was highly immobile when economic risk was perceived as high, coinciding with episodes of economic crisis in the EU (the early 90 s EMS crisis and the 2011 sovereign crisis). However, perfect capital mobility was found when the risk is low. Additionally, financial and trade openness also have an impact on capital mobility. When the openness index was low, we find evidence of moderate capital mobility, while high openness is associated to perfect mobility. This result confirms the relevance of increasing integration in the goods market as a necessary condition to achieve net transfers of financial capital.

In terms of the FH puzzle, we find that the puzzle cannot be rejected if we ignore the structural breaks that are indeed present in our sample. When we allow for structural breaks, the FH coefficient sharply decreases showing high capital mobility, although not completed yet. Our results also show that the FH puzzle tends to return in periods when risk is perceived as high, which may hamper the transmission of monetary policy in the EA.

## Appendix A: Mathematical Transformation of the DCCE into an ECM

1 $$\begin{aligned}&y_{it}=c_{iy}+\phi _{i} y_{i,t-1}+\beta _{0i}x_{it} +\beta _{1i}x_{i,t-1}+\sum ^{\rho T}_{\ell =0} \delta ^{\prime }_{i\ell } \bar{z}_{t-\ell }+e_{yit} \end{aligned}$$

2 $$\begin{aligned}&y_{it}-y_{i,t-1}=c_{iy}+\phi _{i} y_{i,t-1}-y_{i,t-1}+\beta _{0i}x_{it} +\beta _{1i}x_{i,t-1}+\sum ^{\rho T}_{\ell =0} \delta ^{\prime }_{i\ell } \bar{z}_{t-\ell }+e_{yit} \end{aligned}$$

3 $$\begin{aligned}&\Delta y_{it} =c_{iy}-(\phi _{i}-1) y_{i,t-1}+\beta _{0i}x_{it} +\beta _{1i}x_{i,t-1}+\sum ^{\rho T}_{\ell =0} \delta ^{\prime }_{i\ell } \bar{z}_{t-\ell }+e_{yit} \end{aligned}$$

4 $$\begin{aligned}&\Delta y_{it} =c_{iy}-(\phi _{i}-1) y_{i,t-1}+\beta _{0i}x_{it} -\beta _{0i}x_{i,t-1}+\beta _{0i}x_{i,t-1}+\beta _{1i}x_{i,t-1}\nonumber \\&\qquad \quad +\sum ^{\rho T}_{\ell =0} \delta ^{\prime }_{i\ell } \bar{z}_{t-\ell }+e_{yit} \end{aligned}$$

5 $$\begin{aligned}&\Delta y_{it} =c_{iy}-(\phi _{i}-1) y_{i,t-1}+\beta _{0i}\Delta x_{it} +\beta _{0i}x_{i,t-1}+\beta _{1i}x_{i,t-1}+\sum ^{\rho T}_{\ell =0} \delta ^{\prime }_{i\ell } \bar{z}_{t-\ell }+e_{yit} \end{aligned}$$

6 $$\begin{aligned}&\Delta y_{it} =c_{iy}-(\phi _{i}-1) y_{i,t-1}+\beta _{0i} \Delta x_{it}+\gamma _{1i}x_{i,t-1}+\sum ^{\rho T}_{\ell =0} \delta ^{\prime }_{i\ell } \bar{z}_{t-\ell }+e_{yit} \end{aligned}$$

Being: $$\gamma _{1i}=\beta _{0i}+\beta _{1i}$$

7 $$\begin{aligned} \Delta y_{it}=c_{iy}+\beta _{0i}\Delta x_{it}-\lambda _i (y_{i,t-1} -\gamma _{1i}x_{i,t-1})+\sum ^{\rho T}_{\ell =0} \delta ^{\prime }_{i\ell } \bar{z}_{t-\ell }+e_{yit} \end{aligned}$$

$\lambda _i$ and $\gamma _{1i}$ are defined as: $\lambda _i=(1-\phi )$ and $\gamma _{1i}=\frac{\beta _{0i}+\beta _{1i}}{(1-\phi )}$

**Table 14:** KOFEcGI index

Index	Variable name	Variable definitions
KOFEcGI	Foreign direct Investment	Sum of stocks of assets and liabilities of Foreign direct Investment (% of GDP)
	Portfolio investment	Sum of stocks of assets and liabilities of international equity portfolio investments (% of GDP)
	International debt	Sum of inward and outward international portfolio debt securities
		and international bank loans and deposits (% of GDP)
	International reserves	Includes foreign exchange (excluding gold), SDR holdings and reserve position in the IMF (% of GDP)
	International income payments	Sum of capital and labour income to foreign nationals and from abroad (% of GDP)
	Investment restrictions	Prevalence of foreign ownership and regulations to international capital flows
	Capital account openness	Chinn-Ito index of capital account openness
	International investment agreements	Number of Bilateral Investment Agreements (BITs) and Treaties with Investment Provisions (TIPs)
	Trade in goods	Exports and imports of goods (% of GDP)
	Trade in services	Exports and imports of services (% of GDP)
	Trade partner diversity	Average of the Herfindahl-Hirschman market concentration index for exports and imports of goods (inverted)
	Trade regulations	Average of two subcomponents: Prevalence of non-tariff trade barriers and compliance costs of importing and exporting
	Trade taxes	Income from taxes on international trade as percentage of revenue (inverted)
	Tariffs	Unweighted mean of tariff rates
	Trade agreements	Number of bilateral and multilateral free trade agreements

## Footnotes

1 Banking Union and capital markets integration have complementary stability implications for the risk absorption capacity of the Euro Area. While banking integration strengthens the intertemporal risk-sharing channel, which is very effective against temporary shocks, capitals market integration, instead, facilitates the absorption of structural shocks that affect permanent income via risk dispersion and diversification from cross-border holding of assets.

2 The rationale behind this process is that the CMU can speed up the EU recovery from the COVID-19 pandemic. It can also provide the funding needed to deliver on the European Green Deal, make Europe fit for the digital age, and address its social challenges.

3 As peripheral EA countries had fixed their exchange rates with core countries, the needed real exchange rate adjustments could not occur through nominal exchange rate appreciation but took place instead through domestic prices in the periphery.

4 See, among others, Pesaran (2006), Eberhardt and Bond (2009), Chudik and Pesaran (2015) and Ditzen (2018).

5 We follow Dooley et al. (1987) and Lemmen and Eijffinger (1993, 1995) in this section.

6 More recently, alternative measures, investigating the impact of common shocks on prices (*news-based measures*) have been added as a useful complement to the traditional price-based measures.

7 For an in-depth analysis, see Frankel (1988).

8 In contrast, other authors such as Sinn (1992, Jansen (1997, 1998, 2000), Obstfeld and Rogoff (1995), Coakley et al. (1996), and Shibata and Shintani (1998) think that the natural explanation for the existence of a long-run relationship (cointegration) between domestic saving and investment is simply the fulfillment of the long-run solvency condition of the economy and disagree with the conventional FH interpretation. However, this statement seems somehow at odds with the empirical evidence showing persistent long-run trend swings in the FH coefficient in parallel with periods of changes in financial openness.

9 As the literature is very large, we only refer to the most important papers. For a more exhaustive review, see Apergis and Tsoumas (2009) and Coakley et al. (1998).

10 This problem may arise if both variables react to population or productivity shocks or to a government response to correct a current account imbalance.

11 The author called for further research to account for endogenous breaks and heterogeneous parameters.

12 Moreover, their results showed that the FH puzzle was also partially present in the short-run for the majority of northern countries, but not for the periphery, which seems unlikely.

13 ECB (2018) and ECB (2020) analyze how financial integration has evolved in the EA. The ECB measures financial integration using both the price and the quantity-based approaches, concluding that it increased after the creation of the euro with a reversion of the trend after the 2007 financial crisis that stopped after the introduction of the Outright Monetary Transactions (OMT) program and the banking union announcement.

14 Austria (Aus), Belgium (Bel), Denmark (Den), Finland (Fin), France (Fra), Germany (Ger), Greece (Gre), Ireland (Ire), Italy (Ita), Luxembourg (Lux), The Netherlands (Net), Portugal (Por), Spain (Spa), Sweden (Swe) and the United Kingdom (UK). The latter was included since it was part of the EU during the sample period.

15 We have obtained the average index based on the individual indexes from our sample.

16 To be more precise, they are 19 out of 28 countries, that is $68\%$ of its members, $70\%$ after Brexit, as well as $80\%$ of its GDP in 2020.

17 A popular alternative variable to account for global risk is the CBOE Volatility Index (VIX), which measures the stock market’s expectation of volatility implied by the options on the S &P 500 index, calculated and published by the Chicago Board options exchange. However, this variable was not available for the whole period.

18 See Bai and Carrion-i Silvestre (2009) for details.

19 This assumption appears to be unrealistic for the EU and the EA, given the spillover effects. Proof of that is the contagion effect that triggered the Sovereign debt crisis in the EA during the period 2010–2015.

20 CCE can be based on both the MG or Pooled version.

21 The homogeneity assumption of the pool version is not realistic, as it would imply homogeneity for the short-run dynamics and the speed of adjustment, that does not hold from a theoretical point of view.

22 Rejection of the null of a homogeneous long-run coefficient implies using the CS-MG estimator. In contrast, if the null cannot be rejected, the CS-PMG would be a more efficient estimator, since the MG would just add inefficient long-run variability to the estimation.

23 In the case that the break affects the cointegration vector (models 4, 5 and 6), a new long-run variable is added to the regression to take into account the change in the cointegration vector.

24 An alternative for the threshold analysis is the approach developed by Hoke and Kapetanios (2021). However, our sample size is not long enough for its correct implementation.

25 Chudik et al. (2017) also use the CS-ARDL estimator, however, as it was showed by Chudik et al. (2016), the CS-DL estimator has better small sample properties, especially for $T<50$ (as in our case).

26 To clarify, the Chudik et al. (2017) homologous case for our FH model $I=\alpha +\beta S$ would be how the FH relationship would change when a certain $\%$ threshold of Saving/GDP is reached, which is not the goal of this paper.

27 With the exception of the openness index ($T=24$) when it is low.

28 As a robustness check, we have also tried with a spread lower than 100, with no significant differences.

29 With a few exceptions for the *Z* and $Z^*$ tests.

30 Austria, Denmark, France, Germany, Greece, Italy, the Netherlands, Spain, Sweden and the UK.

31 They were three of the eight original members (together with Denmark, France, Germany, Italy and Netherlands).

32 The Soviet Union was one of the most relevant trade partners of Finland, as well as its main supplier of energy. The end of the Soviet Union between 1990–1991 caused a large increase in the cost of energy which was one of the main factors of the Finnish crisis.

33 France, Greece, Ireland, Italy, Portugal and Spain.

34 For the specific case of Finland, the break can be mixing two effects: the Finnish crisis in the early 90 s and the accession to the EU in 1995.

35 The exception being the Netherlands, where the second break is found around the financial crisis.

36 We can reject the null of no cointegration for model 1 both for one and two breaks; however, these are not the best models according to the AIC and BIC criteria.

37 We have found evidence of cointegration for Model 1.

38 With the exception of Belgium, where some short-run impact is observed.

39 We can easily observe discontinuities in the series derived from several relevant economic events that occurred during the period studied: among others, the EMS creation, the signature of the Maastricht Treaty, the inception of the Euro, the financial and sovereign crisis and lastly, the recovery period up to the Covid-19 pandemic.

40 An increase in the risk premium differential means that the 10-year government bond yields are higher for the EA.

41 We have repeated the estimation for a spread lower than 1 (100 basis points) and the result remains unchanged.

42 Probably related with the completion of the Single Market and the elimination of trade and capital controls, as well as the signature of the Maastricht Treaty in 1992.
